# Salivary High-Sensitivity C-Reactive Protein and Its Clinical Relevance in Modern Medicine: A Comprehensive Review

**DOI:** 10.7759/cureus.58165

**Published:** 2024-04-13

**Authors:** Gautam N Bedi, Sourya Acharya, Sunil Kumar, Smruti A Mapari

**Affiliations:** 1 Medicine, Jawaharlal Nehru Medical College, Datta Meghe Institute of Higher Education and Research, Wardha, IND; 2 Obstetrics and Gynecology, Jawaharlal Nehru Medical College, Datta Meghe Institute of Higher Education and Research, Wardha, IND

**Keywords:** high-sensitivity c-reactive protein (hscrp), clinical relevance, diagnosis, inflammation, biomarker, saliva

## Abstract

High-sensitivity C-reactive protein (hsCRP) has emerged as a critical biomarker in inflammation, offering insights into various chronic diseases. However, traditional blood-based assays for hsCRP measurement pose limitations regarding invasiveness and cost. In recent years, saliva has garnered attention as an alternative diagnostic medium, presenting a noninvasive and easily accessible option for biomarker analysis. Salivary hsCRP has thus emerged as a promising avenue for research and clinical application, offering potential advantages over blood-based assays. This comprehensive review aims to elucidate the biological basis of salivary hsCRP, its clinical applications, and methodologies for measurement. By exploring its diagnostic potential, prognostic value, and implications for treatment monitoring, this review highlights the potential impact of salivary hsCRP in modern medicine. Moreover, it emphasizes the need for continued exploration, validation, and integration of salivary hsCRP into routine clinical practice to realize its full potential for enhancing patient care and advancing personalized medicine approaches.

## Introduction and background

High-sensitivity C-reactive protein (hsCRP) is a vital biomarker in inflammation, intricately linked to the acute phase response [1]. Its rapid escalation in response to interleukin-6 (IL-6) stimulation, predominantly from the liver, establishes it as a reliable indicator of systemic inflammation. The significance of hsCRP extends across various chronic conditions, including cardiovascular diseases (CVDs), rheumatoid arthritis (RA), and certain cancers, underscoring its pivotal role in clinical practice and research [2]. Biomarkers have become indispensable tools in modern medicine, offering measurable insights into biological processes and pathological conditions [3]. They aid in disease diagnosis, prognostication, and monitoring treatment efficacy. Among them, hsCRP stands out because it provides valuable insights into underlying inflammatory mechanisms, facilitating informed clinical decision-making and personalized treatment approaches [4].

The exploration of salivary hsCRP represents a compelling proposition driven by the limitations associated with traditional blood-based assays. While conventional methods necessitate invasive and often costly blood sampling, saliva offers a noninvasive, easily accessible alternative for biomarker analysis [5]. This shift in focus toward salivary hsCRP holds promise for overcoming logistical barriers associated with blood sampling, potentially revolutionizing disease detection, monitoring, and management, particularly in scenarios where blood sampling may pose challenges or be undesirable [6]. The objectives of this comprehensive review are multifaceted. Firstly, a comprehensive overview of hsCRP will be provided, elucidating its biological significance and the methodologies employed for its measurement. Secondly, we will underscore the potential advantages afforded by saliva as a diagnostic medium for assessing hsCRP levels, offering insights into its utility in clinical settings. Thirdly, we will delve into the biological underpinnings of salivary hsCRP, including its origins, secretion mechanisms, and factors influencing its levels. Furthermore, it will evaluate its clinical applications across various disease contexts, spanning CVDs, periodontal diseases, and RA. Additionally, it will scrutinize existing methodologies for salivary hsCRP measurement, ranging from conventional techniques to emerging technologies. Lastly, it will assess the clinical implications and relevance of salivary hsCRP in disease diagnosis, prognosis, and treatment monitoring while also identifying future directions, challenges, and opportunities for research and clinical translation in this domain.

## Review

Overview of hsCRP

Definition and Role in Inflammation

Inflammation constitutes a fundamental biological response of bodily tissues to harmful stimuli, such as pathogens, damaged cells, or irritants. This response is characterized by the following five cardinal signs: heat, pain, redness, swelling, and loss of function. The primary objective of inflammation is to eliminate the initial cause of cell injury, clear out damaged cells and tissues, and instigate tissue repair [7]. This protective mechanism involves the coordinated efforts of immune cells, blood vessels, and molecular mediators to combat infections, injuries, or other harmful agents in the body [8]. The role of inflammation is pivotal in the body's defense against infection and injury. Acute inflammation represents the initial response to harmful stimuli, entailing an increased movement of plasma and leukocytes from the blood into the affected area. This acute response is indispensable for healing and typically persists briefly [7,9]. Conversely, chronic inflammation, which may endure for months or even years, entails a prolonged and often deleterious inflammatory reaction that can predispose individuals to various diseases such as asthma, periodontal disease, atherosclerosis, and osteoarthritis [7]. Many factors can instigate inflammation, including microorganisms, physical agents, chemicals, inappropriate immunological responses, and tissue death. Common stimuli for inflammation encompass infectious agents such as viruses and bacteria, physical trauma, burns, radiation injury, and autoimmune disorders [9]. While acute inflammation generally serves a beneficial and transient purpose, chronic inflammation can exert detrimental effects and is linked to a broad spectrum of inflammatory diseases [9].

Measurement Methods and Significance in Clinical Practice

**Figure 1:**
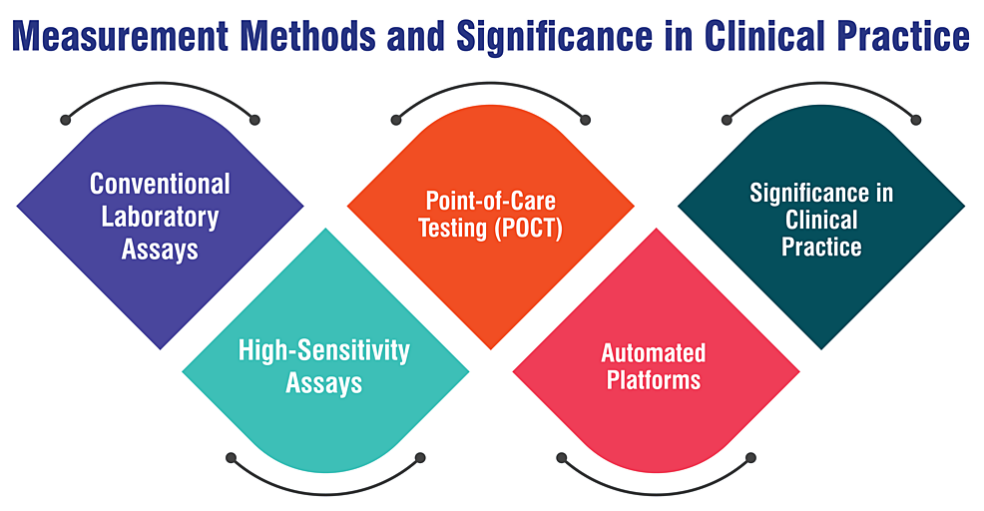
Measurement methods and significance in clinical practice Image Credit: Dr Gautam Bedi

hsCRP is a pivotal biomarker in clinical practice, particularly for assessing cardiovascular risk. To ensure its clinical relevance, diverse methods are employed to measure hsCRP levels accurately. Among these methods is mass spectrometry-based reference measurement procedures, which facilitate the precise quantification of CRP in human serum and plasma, enhancing the accuracy of routine clinical laboratory tests for CRP [10]. Additionally, affinity purification techniques, leveraging monoclonal antibodies against CRP, are employed to isolate CRP from serum proteins, thereby augmenting the feasibility of mass spectrometric quantification [10]. In clinical practice, the measurement of hsCRP assumes significant importance, particularly in cardiovascular risk assessment. Notably, the Cardiophase hsCRP assay plays a crucial role in this domain by quantitatively analyzing exceedingly low levels of CRP in the blood. As a marker for cardiac risk assessment and a prognostic tool in heart disease, this assay significantly enhances risk assessment and therapeutic outcomes in primary CVD prevention [11]. Its integration with lipid evaluation and global risk scoring systems further bolsters risk assessment, benefiting patients with specific risk profiles [11]. Moreover, the clinical relevance of hsCRP measurements is underscored by its pivotal role in stratifying cardiovascular risk. Recommendations advocate for measuring hsCRP in intermediate-risk patients to categorize them into higher or lower-risk groups based on specific hsCRP levels [12]. For instance, hsCRP levels below 1 mg/L indicate lower risk, levels ranging from 1 to 3 mg/L suggest moderate risk, and levels surpassing 3 mg/L indicate a higher relative risk of future heart disease [12]. This stratification empowers clinicians to make informed decisions regarding patient management and the initiation of primary preventive therapy. Measurement methods and significance in clinical practice are shown in Figure 1.

Current Limitations in hsCRP Assessment

**Figure 2:**
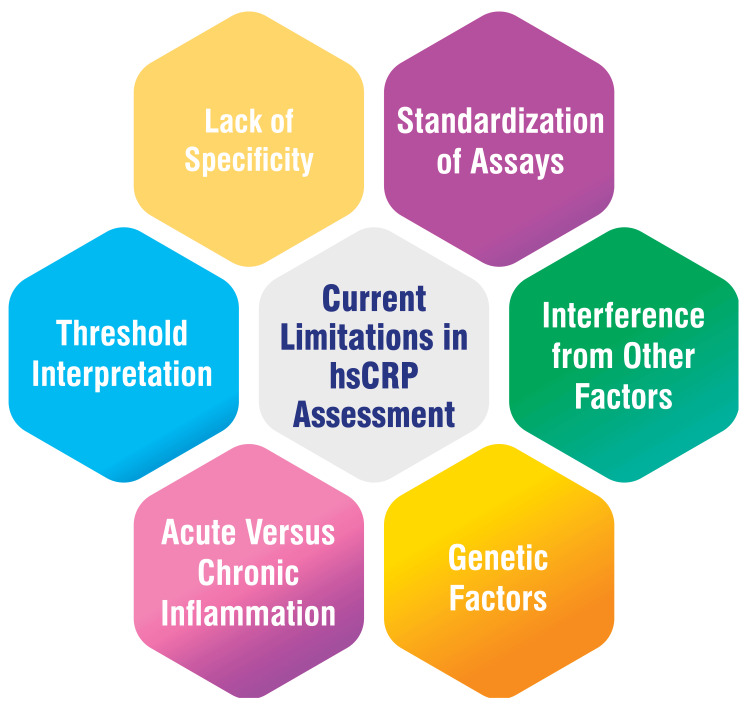
Current limitations in hsCRP assessment Image Credit: Dr Gautam Bedi

Current limitations in assessing hsCRP revolve around the considerable variability observed in hsCRP levels across different populations and the multifaceted influence of various factors on hsCRP concentrations [13,14]. Research indicates notable disparities in hsCRP levels based on age, gender, ethnicity, lifestyle choices, and underlying health conditions [13,14]. For instance, studies have illustrated discernible discrepancies in hsCRP levels between men and women, demonstrating an upward trajectory with advancing age and revealing significant variances among various ethnic groups. Typically, African Americans exhibit the highest hsCRP levels, followed by Hispanics, South Asians, whites, and East Asians [13,14]. This diversity poses challenges in establishing a universal cut-off point for hsCRP, necessitating personalized approaches in interpreting hsCRP levels within clinical contexts [13,14]. Furthermore, the association between hsCRP and CVD risk factors, such as hyperlipidemia and other inflammatory markers, introduces complexity in hsCRP level interpretation for risk assessment [14]. Lifestyle factors, including body mass index, metabolic syndrome, diabetes mellitus, hypertension, oral contraceptive use, physical activity, moderate alcohol consumption, periodontal disease, dietary habits, environmental pollutant exposure, and smoking, also contribute significantly to baseline variation in hsCRP levels [13,14]. This intricate interplay underscores the challenge of solely relying on hsCRP as a standalone marker for predicting CVD risk, highlighting the necessity of considering many factors when using hsCRP for cardiovascular risk assessment [14]. Hence, a comprehensive approach integrating various clinical and lifestyle parameters is essential for accurately gauging cardiovascular risk using hsCRP as a biomarker. Current limitations in hsCRP assessment are shown in Figure 2.

Saliva as a diagnostic medium

Advantages of Saliva Over Traditional Diagnostic Fluids

Saliva is a promising diagnostic medium in modern medicine, offering numerous advantages over traditional fluids. Primarily, saliva collection presents a noninvasive method, facilitating easy and repetitive sampling without necessitating trained personnel, unlike the more invasive blood collection procedures [15,16]. This noninvasiveness minimizes the risk of infection compared to serum sampling and streamlines the sampling process, particularly benefiting individuals such as infants, disabled persons, and those apprehensive about conventional sample collection methods [15]. Furthermore, saliva harbors a diverse array of disease-related biomarkers, rendering it a reservoir of diagnostic information. This rich composition enables the early detection of diseases, thereby furnishing critical insights for effective prevention strategies and tailored treatment interventions [15,16]. Leveraging specific soluble biological markers present in saliva, multiplexed assays can be developed, allowing for the simultaneous detection of multiple biomarkers. These assays can be configured as point-of-care rapid tests or standardized formats suitable for centralized clinical laboratory operations, enhancing diagnostic efficiency and accessibility [16].

Salivary Biomarkers in Clinical Research

Salivary biomarkers play an integral role in clinical research by providing a noninvasive and easily accessible avenue for monitoring health status, disease progression, onset, and treatment outcomes [15,17]. Often referred to as the "mirror of the body," saliva harbors specific biomarkers linked to health and disease, positioning it as a valuable medium for comprehensive health surveillance [18]. The advancement of salivary diagnostic technologies and the identification of salivary biomarkers have significantly bolstered the diagnostic utility of saliva in clinical settings, offering insights into personalized oral healthcare and precision dentistry [17]. Extensive research endeavors have been directed toward identifying salivary biomarkers for various conditions, encompassing infectious diseases such as Zika virus and Helicobacter pylori bacterial infection and oral and systemic disorders [17,19]. Saliva is a promising tool for detecting early symptom onset, facilitating disease monitoring, and tailoring personalized treatment strategies. It underscores its potential to revolutionize disease diagnostics across diverse medical domains [17,19]. In clinical trials, the use of salivary biomarkers has played a pivotal role in elucidating the nuances of personalized oral healthcare and precision dentistry. These investigations have explored the correlation between salivary biomarkers and systemic health, laying the groundwork for real-time measurements beyond the confines of the dental office and their potential integration into daily life [17]. Exploring salivary biomarkers in clinical research offers valuable insights into oral health and furnishes crucial information regarding systemic health conditions, thereby advancing personalized diagnostics and therapy within dentistry [17]. Such endeavors hold immense promise for enhancing disease detection, monitoring, and treatment efficacy, ultimately contributing to improved patient outcomes and quality of life.

Importance of Exploring hsCRP in Saliva

Exploring hsCRP in saliva holds significant promise in modern medicine, driven by saliva's potential as a noninvasive diagnostic medium. Studies have demonstrated a notable positive correlation between salivary and serum hsCRP levels, particularly in conditions such as acute myocardial infarction (MI), thus indicating the viability of saliva as an alternative biofluid for hsCRP assessment in clinical investigations [20,21]. This correlation, observed consistently across MI patients and controls, underscores saliva's diagnostic efficacy in detecting acute MI [20]. The exploration of hsCRP in saliva offers several distinct advantages, including ease of collection, reduced risk of infection, and accessibility across diverse patient demographics, rendering it a valuable tool for disease detection and monitoring [22]. Saliva's versatility as a diagnostic medium is further exemplified by its potential to diagnose various conditions, ranging from infectious diseases to cardiovascular ailments [21]. Moreover, the stability of CRP in saliva at room temperature for up to 8 hours post-collection enhances its utility in both clinical and research settings, ensuring reliable results [22]. Delving into hsCRP in saliva furnishes clinicians and researchers with a noninvasive and dependable method for assessing inflammation and disease risk. The positive correlation between salivary and serum hsCRP levels underscores saliva's potential as a valuable diagnostic fluid, offering novel insights into disease detection, prognosis, and personalized patient management. Such endeavors herald a paradigm shift in diagnostic approaches, emphasizing the transformative impact of saliva-based diagnostics in modern medical practice.

Biological basis of salivary hsCRP

Origin and Secretion of hsCRP in Saliva

The origin and secretion of hsCRP in saliva are intricately linked to its systemic circulation and the body's inflammatory response. The liver synthesizes CRP, an acute-phase protein, in response to inflammation. Within the context of saliva, CRP is thought to enter saliva from the systemic circulation via the gingival crevicular fluid (GCF) [13]. This mechanism suggests that systemic inflammation, as seen in conditions such as acute MI and chronic inflammation, can lead to elevated levels of CRP in saliva. However, local factors within the oral cavity, such as poor oral hygiene, oral inflammation, or diseases, may also influence salivary CRP levels and potentially weaken their association with blood CRP concentrations, mainly when baseline systemic CRP levels are low [13]. Studies have consistently demonstrated a positive correlation between salivary and serum hsCRP levels across various conditions, encompassing MI patients and controls. This suggests that saliva can be an alternative sample for assessing inflammatory markers such as hsCRP [5]. Saliva collection methods, including passive drool and swab-based techniques, offer standardized protocols for sampling and ease of use, rendering saliva a convenient and noninvasive sample for monitoring health status and detecting systemic disorders [5,13]. The secretion of hsCRP in saliva is influenced by systemic inflammation and local oral factors. Saliva thus emerges as a promising alternative biofluid for assessing hsCRP levels, providing valuable insights into the inflammatory status of individuals and offering a noninvasive approach to monitoring health conditions associated with systemic inflammation. This avenue holds significant potential in broadening our understanding of inflammatory processes and improving the diagnosis and management of various systemic disorders.

Factors Influencing Salivary hsCRP Levels

Salivary hsCRP levels are subject to influence from many factors, as underscored by the provided sources. These factors encompass demographic characteristics, environmental variables, health status indicators, lifestyle factors, and seemingly unrelated behaviors such as mobile phone usage [23]. Furthermore, salivary biomarker levels can fluctuate throughout the day, exhibiting variations such as a peak upon awakening and lower levels during daytime hours [5]. The oral cavity environment also significantly influences salivary CRP levels, as CRP is presumed to migrate into saliva from the systemic circulation via the GCF. Local elevations of CRP induced by factors such as poor oral hygiene, oral inflammation, or diseases can potentially distort salivary CRP levels and weaken their correlation with blood CRP concentrations, mainly when baseline systemic CRP levels are low [5]. It is imperative for future studies to meticulously control these influencing factors to ensure the accuracy and reliability of results when measuring salivary hsCRP levels. By diligently accounting for these variables, researchers can enhance their understanding of salivary hsCRP as a potential biomarker for various medical conditions and augment the clinical utility of saliva in modern medicine. Ultimately, such endeavors will refine diagnostic approaches and advance personalized patient care paradigms.

Mechanisms Linking Systemic Inflammation to Salivary hsCRP

The relationship between systemic inflammation and salivary hsCRP involves intricate mechanisms that underscore the interplay between the body's inflammatory response and the presence of hsCRP in saliva. Systemic inflammation, a hallmark of various diseases, stimulates the production of inflammatory markers, including hsCRP. Salivary hsCRP levels serve as a potential reflection of the systemic inflammatory status, providing valuable insights into an individual's overall health condition [24,25]. A pivotal mechanism linking systemic inflammation to salivary hsCRP entails the transfer of minute quantities of CRP from the bloodstream into the salivary glands, primarily facilitated through the GCF [25]. While the liver is recognized as the primary source of CRP production, studies have identified CRP and IL-6 mRNAs within gingival tissue samples, suggesting the potential for local CRP synthesis within the oral cavity. This local production of CRP can significantly influence the relationship between blood and salivary CRP levels [25]. Moreover, the observed association between salivary and serum CRP levels underscores a moderate-to-strong correlation, highlighting the feasibility of using saliva as a noninvasive sample for assessing systemic inflammation through hsCRP measurement [25]. Due to its ease of collection compared to GCF, saliva presents a practical option for the routine assessment of CRP in clinical or field studies. It offers a convenient method for monitoring inflammatory markers such as hsCRP across various conditions, facilitating comprehensive health surveillance and disease management strategies [25].

Clinical applications of salivary hsCRP

Cardiovascular Diseases

CVDs stand as a pressing global health challenge, representing the leading cause of mortality worldwide. Within the realm of CVD research, hsCRP has garnered considerable attention, with studies focusing on its correlation with cardiovascular risk factors and its potential utility as a biomarker for risk assessment [13,26]. Investigations reveal that hsCRP levels exhibit gender disparities, being higher among women than men, and escalate with advancing age, with notable variability observed among diverse populations and ethnicities [13]. The association between hsCRP levels and established CVD risk factors such as hyperlipidemia, diabetes mellitus, and hypertension underscores the intricacies inherent in using hsCRP as a universal risk indicator [26]. In the clinical arena, the integration of hsCRP into CVD risk prediction remains a subject of ongoing research and debate. While certain studies propose a modest correlation between hsCRP levels and the risk of coronary heart disease post-adjustment for conventional risk factors, the optimal clinical application of hsCRP in risk prediction lacks unanimous consensus [13,26]. Moreover, extending hsCRP's utility in risk assessment beyond conventional parameters and establishing a standardized threshold for hsCRP levels continue to elicit discourse within cardiovascular medicine [13]. Overall, the role of hsCRP in CVDs underscores the imperative for personalized approaches in interpreting hsCRP levels, accounting for variables such as lifestyle choices, ethnicity, and additional inflammatory markers. Further research endeavors are essential to comprehensively elucidate the clinical ramifications of hsCRP in CVD risk prediction and to delineate its precise role in tailored patient management strategies. This concerted effort will be instrumental in refining risk assessment protocols and advancing personalized care paradigms in cardiovascular medicine.

Periodontal Diseases

Periodontal diseases, commonly called gum diseases, encompass a spectrum of inflammatory conditions impacting the supportive structures surrounding the teeth, including the gums, bone, and periodontal ligament. These diseases contribute to tooth loss and play a role in systemic inflammation [27]. The onset and progression of periodontal disease involve a disruption of the oral microbiota, triggering interactions with the host's immune system and ultimately leading to inflammation and disease manifestation [27]. This pathophysiological cascade persists through periods of activity and dormancy until the affected tooth is extracted or the microbial biofilm is effectively removed, allowing inflammation to subside [27]. The severity of periodontal diseases is influenced by a myriad of environmental and host-related risk factors, encompassing both modifiable factors such as smoking and non-modifiable factors such as genetic susceptibility [27]. Prevention strategies primarily revolve around daily oral hygiene practices and regular professional removal of microbial biofilm, typically quarterly or bi-annually [28]. Emerging treatment modalities include antimicrobial therapy, host modulation therapy, laser therapy, and tissue engineering aimed at tissue repair and regeneration [28]. Periodontal diseases present complex challenges characterized by inflammation affecting the structures supporting the teeth. A comprehensive understanding of the microbial and immune interactions involved in these diseases is pivotal for the development of effective prevention and treatment strategies, emphasizing the significance of promoting oral hygiene practices and ongoing exploration of innovative therapeutic interventions.

Rheumatoid Arthritis

RA stands as a chronic inflammatory disorder characterized by the immune system's assault on the tissue lining the joints, leading to symptoms of pain, swelling, and joint damage. Commonly affecting joints in the hands, wrists, and knees, RA manifests with joint pain, tenderness, stiffness, and morning stiffness lasting 30 minutes or more [29-31]. Beyond joint involvement, RA can extend its impact to other organs, such as the eyes, heart, lungs, and blood vessels. While the precise cause of the disease remains elusive, it is widely believed to result from a combination of genetic predisposition and environmental triggers, prompting the immune system to erroneously target healthy cells, notably the synovium lining the joints [31,32]. Diagnosis of RA entails a comprehensive assessment, including a detailed medical history, physical examination, and specific blood tests such as erythrocyte sedimentation rate and C-reactive protein (CRP) levels to detect inflammation and RA-associated antibodies. Timely diagnosis is paramount in initiating effective treatment to forestall long-term joint damage and deformities [31,32]. Treatment modalities for RA encompass disease-modifying antirheumatic drugs (DMARDs) and biological response modifiers aimed at impeding disease progression, curbing inflammation, and averting joint deformities. Lifestyle adjustments such as regular physical activity, weight management, and smoking cessation play pivotal roles in RA management, fostering improved quality of life [32]. RA exerts significant physical and social repercussions, disrupting daily activities, work, and overall well-being. Effective management necessitates a multidisciplinary approach, integrating specialized care from rheumatologists, tailored physical activity regimens, and self-management strategies aimed at alleviating pain, minimizing disability, and mitigating disease-related complications [31]. Through concerted efforts, individuals with RA can achieve enhanced symptom control, improved function, and a better overall quality of life, emphasizing the importance of comprehensive and holistic care in managing this chronic inflammatory condition.

Other Potential Applications and Emerging Research Areas

Salivary CRP has emerged as a promising biomarker for diabetes monitoring, with studies revealing a positive correlation between salivary and serum CRP levels in individuals with type 2 diabetes (T2D) [21]. This correlation underscores the potential of salivary CRP as a noninvasive tool for tracking diabetes progression and assessing disease severity. The utility of salivary CRP extends beyond diabetes to encompass systemic and oral disorders, where it has shown promise as a biomarker for various medical conditions [23]. Its application in disease assessment, diagnosis, monitoring, and therapeutic interventions underscores its versatility in clinical practice, offering valuable insights into disease pathology and progression. Research has also identified a link between elevated salivary CRP levels and cardiovascular risk behaviors, particularly about active and passive smoking among healthy youth [33]. This association suggests the potential utility of salivary CRP as a noninvasive biomarker for assessing inflammatory pathways linked to cardiovascular risk factors, thereby facilitating early intervention and risk mitigation strategies. Furthermore, salivary CRP holds promise as a tool for global risk assessment, emphasizing the importance of understanding population distributions of hsCRP and its clinical implications [34]. This highlights the potential of salivary CRP in providing valuable insights for evaluating overall disease risk within populations, thus contributing to more targeted and effective public health interventions.

Methodologies for salivary hsCRP measurement

Conventional Techniques

Conventional methods for measuring salivary hsCRP typically employ enzyme-linked immunosorbent assays (ELISA) tailored explicitly for saliva samples. These ELISA kits are finely tuned for human salivary research, providing a dependable means to measure hsCRP levels in saliva with accuracy and precision [35,36]. The "passive drool" technique is commonly employed for saliva collection, allowing for straightforward sampling by healthcare professionals or individuals without medical expertise [37]. Saliva can be collected through various means, such as spitting, chewing, or swab-based techniques. The chewing is often preferred for eliciting stimulated saliva from the parotid glands, which is recommended for measuring CRP concentration in saliva [37]. The ELISA kits for measuring salivary hsCRP adhere to an indirect sandwich ELISA principle. This methodology entails the capture of CRP using pre-coated antibodies on a plate, followed by detecting anti-CRP antibodies linked to enzymes such as horseradish peroxidase. This enzymatic reaction yields color changes that are quantified using a standard plate reader, ensuring precise and standardized assessment of hsCRP levels in saliva [35,37].

Novel Approaches and Technologies

Innovative approaches and emerging technologies in salivary biomarker research are reshaping the landscape of noninvasive diagnostic tools for various health conditions. One such advancement involves using microchip assay systems designed for measuring CRP levels in human saliva, aiming to address the challenges posed by the typically lower concentrations of salivary constituents compared to serum [38]. These microchip systems present a promising avenue for point-of-care diagnostics using saliva, potentially revolutionizing noninvasive diagnostic testing practices. Additionally, cutting-edge techniques such as lab-on-a-chip systems are being investigated for their potential to enhance the sensitivity and specificity of detecting inflammatory markers in saliva [37]. While these technologies encounter hurdles related to the high viscosity and heterogeneous properties of saliva, ongoing advancements can significantly enhance the detection and quantification of salivary biomarkers associated with various systemic disorders. Furthermore, the development of wearable modules designed for automated saliva collection, alongside the establishment of standardized protocols for oral sampling, contributes to the convenience and reliability of obtaining saliva samples for biomarker analysis [37]. These advancements streamline the collection process and open doors for personalized and proactive healthcare applications that harness salivary biomarkers for early disease detection and continuous monitoring. By leveraging these innovative approaches and technologies, researchers and healthcare professionals are poised to enhance diagnostic capabilities, ultimately facilitating more effective disease management and improved patient outcomes.

Challenges and Considerations in Assay Development

Developing assays for measuring salivary hsCRP entails navigating several challenges and considerations researchers must carefully address. One significant challenge lies in the intricate nature of saliva as a biofluid, comprising a mixture of fluids originating from various microenvironments such as gingival crevices. This heterogeneity can affect the consistency and accuracy of salivary biomarker measurements, emphasizing the critical need for standardization of collection, handling, and storage procedures to ensure reliable results in salivary analysis [37]. Another crucial consideration in assay development is the necessity for noninvasive methods to quantify hsCRP levels in saliva. While saliva presents a convenient and accessible alternative to serum for biomarker analysis, establishing robust and sensitive assays tailored for salivary hsCRP measurement is imperative for clinical applicability [35]. The advent of ELISA designed explicitly for saliva samples, such as the Salimetrics® Salivary C-Reactive Protein ELISA Kit, has played a pivotal role in facilitating accurate and quantitative assessment of hsCRP levels in saliva [35]. Moreover, interpreting salivary CRP levels about systemic CRP levels poses a notable challenge in assay development. Gaining insights into the relationship between salivary and serum CRP, including factors such as local CRP production within the oral cavity and the impact of oral health status on CRP levels, is essential for the precise interpretation of salivary hsCRP measurements in both clinical and research settings [35,39]. Addressing these challenges and considerations is fundamental to advancing the development of assays for salivary hsCRP measurement and enhancing their clinical utility in disease diagnosis, monitoring, and management.

Clinical relevance and implications

Diagnostic Potential of Salivary hsCRP

Salivary hsCRP emerges as a clinically significant marker in modern medicine, notably acute MI and chronic inflammation. Studies have revealed a positive correlation between salivary and serum hsCRP levels in MI patients. This suggests saliva's potential as a noninvasive alternative for assessing hsCRP levels and offering diagnostic value in detecting acute MI [13,20]. Additionally, using salivary biomarkers, including hsCRP, presents a novel and minimally invasive approach for diagnosing chronic inflammation, thereby providing clinicians with innovative avenues for tailored patient management [40]. The clinical relevance of salivary hsCRP extends to conditions such as late-onset neonatal sepsis, where research has explored salivary CRP as a potential substitute for serum screening, albeit with inconsistent findings [34]. While serum CRP remains a standard diagnostic tool, debates surrounding its accuracy in late-onset sepsis prompt considerations for the usefulness of salivary CRP as a diagnostic marker for neonatal pneumonia [34]. In CVD, hsCRP has been extensively investigated, revealing associations with CVD risk factors and underscoring the complexity of its utility as a universal risk marker [37]. While elevated hsCRP levels have been linked to increased CVD risk, the optimal clinical application of hsCRP in risk prediction is subject to ongoing research and debate [37]. The variability in hsCRP levels among diverse populations and the influence of lifestyle factors emphasize the necessity for personalized approaches when interpreting hsCRP levels in clinical practice [37]. The clinical significance of salivary hsCRP in modern medicine underscores saliva's potential as a noninvasive diagnostic tool for various conditions, providing insights into acute MI, chronic inflammation, and neonatal sepsis. Further research is imperative to comprehensively elucidate the clinical implications of salivary hsCRP in disease diagnosis, prognosis, and personalized patient management.

Prognostic Value and Predictive Utility

Salivary hsCRP emerges as a crucial prognostic marker and predictive tool in modern medicine, particularly acute MI and chronic inflammation. Research indicates a positive correlation between salivary and serum hsCRP levels in MI patients, suggesting saliva's potential as a noninvasive alternative for assessing hsCRP levels [20,21]. This correlation, observed in MI patients and controls, underscores saliva's diagnostic utility in detecting acute MI [21]. Salivary hsCRP is a pivotal biomarker for T2D screening, emphasizing its predictive value in identifying disease markers and gauging disease progression [21]. Moreover, comparative assessments of salivary and serum hsCRP in acute MI patients yield promising results, demonstrating a positive correlation between salivary and serum hsCRP levels in both MI patients and controls [20]. This correlation underscores saliva's potential as an alternative fluid for assessing hsCRP levels and highlights its predictive utility in diagnosing acute MI and monitoring disease progression [20]. In the broader context of salivary biomarkers, including hsCRP, their diagnostic utility is recognized across various conditions, such as CVDs, systemic inflammation, and metabolic disorders [37]. Saliva's noninvasive collection method and ability to reflect systemic conditions make it an attractive option for predicting and monitoring disease states. It provides a valuable tool for personalized patient management and early detection of chronic diseases [37]. Therefore, salivary hsCRP serves as a prognostic indicator for conditions such as acute MI and T2D and demonstrates predictive utility in assessing disease progression, monitoring treatment efficacy, and potentially revolutionizing diagnostic practices in modern medicine.

Monitoring Disease Progression and Treatment Efficacy

Assessing biomarkers such as hsCRP in both serum and saliva can facilitate the monitoring of disease progression and treatment efficacy. Research has unveiled a positive correlation between salivary and serum hsCRP levels in conditions such as acute MI, underscoring saliva's potential as a noninvasive alternative for assessing hsCRP levels [20]. This correlation, observed in both MI patients and controls, underscores the diagnostic utility of saliva in detecting acute MI [20]. Saliva emerges as an alternative biofluid for hsCRP determination in MI patients, providing a noninvasive tool for clinical investigations [20]. Moreover, studies have demonstrated the effectiveness of nonsurgical periodontal therapy in influencing salivary biomarkers, including CRP, in individuals with T2D and non-diabetic generalized chronic periodontitis. Non-surgical periodontal therapy has significantly reduced CRP levels in diabetics and non-diabetics, suggesting its potential role in lowering crucial salivary biomarkers and monitoring disease progression [41]. As a noninvasive diagnostic tool, saliva can be critical in monitoring glucose levels in p with T2D and chronic periodontitis, offering a valuable approach to disease management and treatment evaluation [41].

Integration into Routine Clinical Practice

Integrating salivary hsCRP into routine clinical practice holds significant promise in modern medicine, particularly concerning conditions such as diabetes and CVD. Saliva, a noninvasive biofluid, offers a convenient and accessible medium for measuring biomarkers such as CRP, presenting a valuable alternative to traditional blood-based testing [21,42]. In clinical settings, the use of salivary CRP has demonstrated positive correlations with serum CRP levels, showcasing its potential as a diagnostic tool for conditions such as diabetes. Salivary CRP concentration has been associated with low-grade inflammation linked to T2D, highlighting its relevance in disease screening and monitoring [21]. The noninvasive saliva collection, especially from children, enables repetitive sampling, aiding in early detection, prognosis, and therapy monitoring for various diseases, including diabetes [21]. Furthermore, the diagnostic accuracy of salivary CRP in predicting serum CRP thresholds underscores its clinical utility. Research indicates that raw and protein-adjusted salivary CRP concentrations exhibit good diagnostic accuracy in predicting serum CRP levels, rendering salivary CRP a valuable tool for disease screening and risk assessment [42]. The normalization of salivary samples using total protein concentration enhances the reliability of salivary CRP measurements and their correlation with serum CRP levels [42]. Incorporating hsCRP, including salivary CRP, into risk prediction models has garnered interest in the broader context of CVD. While the variability of hsCRP levels among different populations and the influence of lifestyle factors present challenges, the potential of hsCRP as a biomarker for personalized risk assessment in CVD continues to be a subject of ongoing research and discussion [13,43]. Considering hsCRP levels alongside traditional risk factors such as LDL-C has shown improved prognostication, highlighting the potential of hsCRP in refining risk assessment strategies [34].

Future directions and challenges

Opportunities for Further Research and Validation

**Figure 3:**
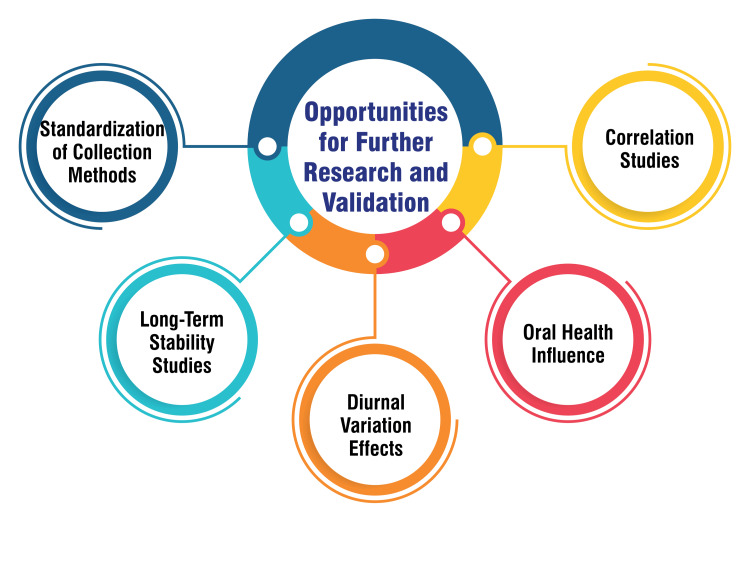
Opportunities for further research and validation Image Credit: Dr Gautam Bedi

Research efforts should prioritize standardizing collection methods for saliva samples to optimize the accuracy and reliability of salivary CRP measurements. Establishing standardized protocols should encompass considerations such as salivary flow rate and storage conditions to enhance result consistency [5]. Investigations into the long-term stability of salivary CRP levels are paramount for comprehending the reliability of saliva as a diagnostic tool over an extended period. Research should delve into the stability of CRP in frozen saliva samples, extending beyond current knowledge limited to short-term stability assessments [5]. Further studies are warranted to evaluate the impact of diurnal variations on salivary CRP levels. Understanding the fluctuations of CRP levels throughout the day and their potential influence on diagnostic accuracy is essential for accurate result interpretation [44]. Exploration into the effects of oral health conditions on salivary CRP levels is imperative. Investigating how factors such as oral hygiene, inflammation, or oral diseases can affect salivary CRP concentrations will elucidate the relationship between salivary and serum CRP levels [45]. Conducting larger-scale studies is necessary to establish a robust correlation between salivary and serum hsCRP levels, particularly in specific clinical conditions such as acute MI. Correlational studies between salivary CRP and serum CRP levels across diverse patient populations will validate the utility of saliva as an alternative fluid for assessing hsCRP levels [20]. Opportunities for further research and validation are shown in Figure 3.

Ethical Considerations and Patient Privacy

Ethical considerations and patient privacy are paramount in modern medical practices when using sensitive biomarkers such as salivary hsCRP. Health professionals are bound by legal and ethical obligations to uphold patient confidentiality, foster trust, and safeguard patient safety [46]. Any breaches of confidentiality, whether stemming from negligence or malicious intent, can potentially undermine the physician-patient relationship and jeopardize patient well-being [46]. Therefore, maintaining confidentiality is essential for preserving patient privacy and upholding the integrity of healthcare practices. In using salivary biomarkers such as hsCRP, ensuring patient privacy and data security is paramount. Collecting, storing, and analyzing salivary samples containing sensitive health information necessitate strict adherence to ethical guidelines and data protection laws to safeguard patient confidentiality [47]. Standardized collection methods and protocols are crucial in mitigating the risk of unauthorized disclosure and protecting patients' health data [47]. Additionally, healthcare professionals must possess a comprehensive understanding of ethical regulations, data security statutes, and confidentiality protocols to responsibly manage patient information and maintain the trust of both patients and society [46]. Continuous training in medical ethics for healthcare personnel is indispensable for reinforcing the significance of patient confidentiality and privacy in medical practice [46]. By prioritizing ethical considerations and patient privacy, healthcare providers can uphold the core tenets of patient-centered care and ensure the ethical application of sensitive biomarkers such as salivary hsCRP in clinical contexts.

Overcoming Barriers to Adoption in Clinical Settings

Addressing barriers to integrating salivary hsCRP in clinical settings necessitates overcoming sample collection and standardization challenges. A significant hurdle is the need for a universally accepted method for collecting saliva samples to optimize salivary CRP levels for monitoring systemic inflammation [47]. Consistent collection techniques contribute to varying dilution within the oral cavity, affecting salivary CRP measurements' precision [47]. Research efforts must establish standardized protocols that regulate factors such as salivary flow rate, stimulation level, collection site, and methodology to enhance the accuracy and reliability of salivary CRP as a diagnostic tool [47]. Ensuring precise control over the fluctuating flow rate of saliva is critical for accurate salivary CRP measurements. Various factors, including stimulation levels, medications, and comorbid conditions such as diabetes, can influence salivary flow rate, leading to intra-individual variability [48]. Although several methods have been developed to manage salivary flow rate, there have yet to be definitive solutions, underscoring the necessity for further investigation in this domain [48]. Furthermore, endeavors to standardize analyte levels by assessing total salivary volume or protein concentration may necessitate large sample volumes, posing practical challenges for point-of-care devices and clinical applications [48]. To facilitate the incorporation of salivary CRP into clinical practice, it is imperative to surmount these obstacles by establishing uniform protocols for sample collection, effectively regulating salivary flow rate, and validating the correlation between salivary and serum CRP levels. By overcoming these challenges, salivary hsCRP could emerge as a valuable instrument for monitoring inflammation and disease progression in a noninvasive and convenient manner, thereby transforming inflammation management in clinical settings [47].

## Conclusions

In conclusion, this comprehensive review has thoroughly examined salivary hsCRP and its potential impact on modern medicine. Through exploring its biological significance and clinical applications, valuable insights have been gained into the role of this noninvasive biomarker in disease diagnosis, prognosis, and treatment monitoring. Salivary hsCRP holds promise as a convenient and accessible tool for assessing systemic inflammation, potentially revolutionizing clinical practice by offering a less invasive alternative to traditional blood-based assays. Moving forward, continued exploration and use of salivary hsCRP are essential. Further research is needed to validate its diagnostic accuracy and prognostic value, while efforts to standardize assay methodologies and integrate salivary hsCRP into routine clinical practice should be prioritized. By collectively advancing our understanding and use of salivary hsCRP, we can unlock its full potential to improve patient care and drive advancements in modern medicine.
